# Setting up a structured laboratory mentoring programme

**DOI:** 10.4102/ajlm.v2i1.77

**Published:** 2013-03-26

**Authors:** Talkmore Maruta, Philip Rotz, Trevor Peter

**Affiliations:** 1 Clinton Health Access Initiative, Lesotho

## Abstract

**Introduction:**

Laboratory mentoring programmes can be an important vehicle to establish and solidify quality management systems and help laboratories achieve accreditation goals. Different mentoring approaches have been used with varying levels of success. The authors provide a guide to implementing a structured laboratory mentorship programme based on their practical field experience.

**Method:**

The study is based on experience in Lesotho as well as subsequent roll out of a similar approach in the other African countries of Zimbabwe, Mozambique, Swaziland and Cameroon between 2009 and 2011.

**Summary:**

We highlight critical elements to consider when setting up a long-term, sustainable and well-structured mentorship programme. These elements include: well-defined goals; sufficient length of mentor engagement on site; standardised approach across laboratories; measurement of progress using standardised tools; well-structured reporting mechanisms; alignment of the programme with overall Ministry of Health plans; and selection and training of the mentors. These elements will differ in application, depending on countries’ needs and available resources. A structured approach allows for scalability, comparison across laboratories and countries and an easier approach to budgeting and planning for countries intending to set up similar programmes.

## Introduction

In recent years, attention has increasingly been turning towards improving overall healthcare in order to ensure higher quality, greater access and better value for money.^1^ To achieve this, countries have used various approaches, mainly training health professionals.^1^ One approach has been introducing quality improvement and quality management principles and concepts into pre-service or in-service training curriculum.

Quality-related curriculum in most medical trainings has been under-emphasised, despite frequent recommendations.^2^ In a survey conducted by the Health Foundation in 2012,^1^ quality improvement was found to be mandatory for pre-service medical training only in the USA. This leaves much of the training in quality to the in-service period for most other countries. In-service training ensures that health professionals who are already providing services have the opportunity to update their knowledge and skills according to the latest standardised practices. Many different approaches have been used in quality related in-service training of health professionals, including classroom-based training, online training, workshops, and single session continued medical education courses.

An interactive training approach that provides competency-based training has been shown to be effective in transferring skills as well as knowledge and attitudes.^3^ A culture of practicing quality routinely is a behaviour change issue and calls for a different approach to training.^4^ The Foundation of Health survey indicated that approaches that utilise active learning strategies, where participants put quality improvement into practice through close coaching and mentoring, are thought to be more effective than didactic classroom styles alone.^1^

However, most of the documented in-service training models are largely for clinical practice. Anecdotal reports suggest that the laboratory side of healthcare has also been active in this area, but that most of the work remains undocumented. One successful model that has been documented is the Lesotho mentorship model^4^, where a facility based approach by an experienced mentor resulted in considerable transfer of quality management skills to the laboratory staff.

Based on practical field experience from the Lesotho mentorship model^1^ and subsequent roll out of similar models in five other African countries (Mozambique, Zimbabwe, Swaziland, Cameroon, and Nigeria) as well as selected laboratories under the East African Public Health Laboratory Networking Project (EAPHLNP), we give guidance on how to set up a structured laboratory mentorship programme. The facility-based approach, with mentors spending extended periods of time on site, was also documented by Gershy-Damet et al.^5^ who recommended structured periods of mentor engagement times embedded in the daily life of a laboratory as valuable means for successful implementation of quality management systems.

## Facility-based laboratory mentorship

Laboratory mentoring programmes can be an important vehicle to establish and solidify quality management systems (QMS) and help laboratories achieve accreditation goals.^1^ There are other methods that have been used to assist laboratories in attaining accreditation. The method described here is an initiative where experienced mentors were embedded in a laboratory for extended periods of time to develop an in-depth understanding of the laboratory and provide day-to-day assistance in the implementation of QMS and preparation for accreditation. This facility-based and embedded approach to mentorship has been observed to have high impact in different settings.^4,5^

The authors recognise that mentoring can be utilised for a variety of laboratory support purposes. For example, mentoring has proven to be a useful means of establishing new tests, like DNA-PCR, viral load and TB culture. In these initiatives, an experienced mentor works with a laboratory for several months to help set up and problem-solve through the initial rollout of a given test. The mentoring approach discussed in this document engages the whole laboratory, targets QMS implementation throughout the entire testing system and is staged as a series of engagements that aid laboratories as they progress toward accreditation.

There are several training tools available in QMS implementation.^6^ Mentoring can be used independently or in conjunction with these tools, One of the tools that has been shown to have demonstrable, documented evidence of improvements is the Strengthening Laboratory Management Toward Accreditation (SLMTA) training initiative, a task-based laboratory management training developed jointly by the United States Centers for Disease Control and Prevention, the American Society for Clinical Pathology, and the Clinton Health Access Initiative.^7^ In some settings, SLMTA and mentoring have been shown to have better impact than each implemented independently.^8^ Whether using mentoring as a means to deliver SLMTA at the laboratory level or as second-level support for laboratories that have completed SLMTA, these two efforts complement one another and can strengthen the drive to realise national accreditation goals.

Described below are some of the critical building blocks to consider when setting up a facility-based laboratory mentorship programme. The authors recognise that these suggested building blocks may not always apply in the same way in every setting, but believe that each of these elements should be considered. For example, although time spent on site is a critical element, the amount of time suggested in this guide may not be feasible in all cases.

## Identifying and training mentors

Special attention must be taken when selecting mentors, as they are a critical element in the success of the mentorship program.^4^ Mentors can be drawn from experienced laboratory personnel available internationally, regionally or nationally. International mentors with experience in preparing laboratories for accreditation may provide valuable laboratory mentoring support. However, these persons can be difficult to identify, costly to employ and may not be available for extended periods of time. Building a mentoring programme around regional and national mentors should be seriously considered. When compared to the cost of international mentors, the same level of funding may employ a greater number of regional or national mentors and therefore provide more total contact time with laboratories.

In addition, while carefully selected international mentors may have experience that enables them to contribute particular expertise, it should also be recognised that experience with QMS implementation under conditions common to many African laboratory systems is also an expertise – one well-suited to mentoring laboratories for accreditation. In addition, the in-depth familiarity with regional laboratory practice and national laboratory systems possessed by regional and national mentors can also contribute significantly to mentoring efficiency and effectiveness. Further, investing resources in the development of regional or national mentors is a means of building capacity and strengthening long-term sustainability.

One of the criteria to consider when selecting mentors is whether they have broad experience as a bench technician. This is important, as the mentor works in an embedded fashion. Some management experience, at least at the bench section level, is also an important element to be considered, as the mentor would deal with varying management related issues. Some background knowledge of QMS and implementation of ISO laboratory standards is vital to ensure that whatever is implemented and the resolving of non-conformities remain aligned with international standards. Experience in conducting training would be useful, as training is one of the techniques used in delivering this facility-based mentorship.^4^

Mentoring involves behaviour change. Hence it is desirable to have a mentor who is persistent, patient and has a positive attitude, coupled with passion for laboratory quality improvement. The ability to work well with others and the ability to provide instruction and firm constructive correction is essential.

Not all the elements described above might be present in all potential candidates for mentoring. Some elements, such as knowledge of standards, can be attained after recruitment through training and attachments at accredited laboratories or those working towards accreditation. This can provide the much needed hands-on experience in implementation of QMS.

Once mentors have been selected, preparatory training will aid in readying new mentors for deployment. Experienced mentors should be sought to provide training for new recruits. Their training should include some didactic training, job-shadowing, working under the observation of an experienced mentor, work plan sharing and feedback with experienced mentors or amongst a pool if all are new. On-site follow-up reviews, especially during the early phases of the mentorship, are one means of training new recruits.

In addition to this training, there are several additional training courses or experiences which may prove beneficial for mentors, either as part of their initial preparation or as ongoing professional development. Examples include SLMTA training-of-trainers workshops and ISO 15189 laboratory standard training. Training in conducting laboratory assessments should be considered, as assessments are an integral component of the mentoring described.

In countries where resources allow, international and/or regional mentors are used. In such cases, developing local capacity must be prioritised from the beginning of the programme. Several methods are suggested in ensuring building of local mentoring capacity within the mentorship programme. Local experienced laboratory staff can be twinned with an experienced internal or regional mentor. Short- to long-term attachments at mentored laboratories or accredited laboratories where available, are one way of building local mentoring capacity. Structured exchange programmes between laboratories, where peer-to-peer training and skills exchange can groom potential local mentors, are a cost-effective means of training and grooming local mentors.

## Time: An essential resource commitment

Mentoring programmes require resource commitment. The resource most critical to effective mentoring is time.^4^ Mentors spend extended periods of dedicated time working with laboratories in an embedded fashion – an investment of weeks to months of in-laboratory presence. Side-by-side instruction and guidance, provided through sustained time in the laboratory, are essential to create a culture that understands and values quality and to effect behaviour changes within the laboratory that ensure routine implementation of quality measures. It is worth noting that the side-by-side mentorship approach does not target technical skills training. Emphasis is on assisting the mentees to build quality into the testing that they are already competent on.

With adequate resource commitment, a well-structured mentoring programme can equip and empower laboratory staff and bolster laboratory performance. However, not all countries or initiatives are able to commit the in-lab staff time necessary for an effective mentoring programme. Where resource commitment to mentoring is not currently feasible, consideration should be given to what can be accomplished through a programme of shorter duration coaching and/or site support visits.

## Optimal environment for mentoring success

Every effort should be made to ensure that mentoring programmes are implemented within a context conducive to the realisation of meaningful and sustainable laboratory improvement. Mentoring efforts that are established without attention to the environmental conditions of the laboratory system are likely to find their success limited and short-lived.

Ministry of Health (MOH) support and involvement is crucial to mentoring success. MOHs should be clear in their communication of accreditation goals, and mentoring as a way of building the knowledge, capacity and experience necessary for success. Public identification of mentoring as an MOH priority can increase the acceptance and standing of mentors in the eyes of laboratory staff and hasten buy-in and cooperation.

It is advisable to detail programme expectations and responsibilities in a document shared with all parties. This encourages clarity, transparency, commitment and accountability. In some countries, this has even taken the form of a ‘contract’ between the MOH, the laboratories, the mentors and the organisation they represent.

Programme design and site selection should be done in consultation with MOH laboratory services leadership and in line with national accreditation goals detailed in laboratory strategic and operational plans. Further, mentoring programmes should be designed with direct links to national laboratory quality assurance (QA) programmes. Mentors should report and route information to the QA office and act as an extension of the QA office at the laboratory level, assisting with in-lab implementation of national QA initiatives.

An added advantage for mentoring programme implementation is the presence of a national laboratory technical working group or a subcommittee dedicated to implementation of QMS or accreditation. Mentoring programmes should be represented in such a group to encourage sharing of on-the-ground challenges with a wider group of stakeholders and collective solution seeking.

Lastly, the optimal context for mentoring will also include active parallel national strengthening initiatives for service and maintenance of instruments, supply chain, proficiency testing, and routine assessment of laboratory performance. In-lab mentoring can yield many service-level benefits, but a number of critical areas may require action at the management level, far removed from any individual laboratory. Combining laboratory mentoring efforts with initiatives that strengthen the national laboratory system provides the best opportunity for long-term success.

## Structured mentoring model

Mentoring programme design should be based upon well-defined goals and the model developed should reflect the following key commitments:

- Mentors spend extended periods of time in laboratories.
- Mentors are embedded in the life of the laboratory.
- Mentoring is delivered through a series of engagements over time.
- Laboratory progress is measured at specific points with standard tools (e.g., WHO-AFRO Strengthening Laboratory Quality Improvement Process Towards Accreditation [SLIPTA] checklist).
- Reporting is structural.

The mentoring programme design will reflect the conditions and resources particular to each context. While mentoring programmes will differ, care should be taken to maintain the commitments detailed above.

Mentoring is about embedding someone in the life of a laboratory to fully understand its culture, processes and people. Mentors work from within and alongside to help raise a laboratory’s level of performance. The authors have observed that mentorships of longer than four to eight weeks can be more effective than shorter periods.^4^

Though special mentoring emphasis may be given to laboratory staff with greater levels of responsibility, facility-level mentoring should involve all laboratory staff and not centre solely on the manager or quality officer. Mentors should emphasise a team approach to quality. The entire facility should have a stake in the improvement of the laboratory.

Several common variables should be considered in designing a mentoring model that will help laboratories achieve accreditation goals:

### Sufficient duration

Mentoring requires a sustained time commitment. Significant periods of contact time with the laboratory are necessary to change behaviour and practice. The initial engagement is crucial to success. It is during this time that mentors develop a comprehensive understanding of the rhythms, patterns, practices, and personalities within the laboratory. This period is also essential for relationship building, as many positive changes are realised as much through common cause as particular expertise. Initial mentoring engagements of less than four weeks may not achieve this. Some programmes dedicate a mentor to a laboratory for six to twelve consecutive weeks for an initial engagement.

### Sufficient frequency of mentor engagement

We view laboratory mentoring as a series of mentoring engagements. This approach enables the mentor to assist in implementation and contains periods where the laboratory functions on its own initiative. A model that prioritises several engagements with a given laboratory, in our view, gives the mentor and MOH leadership the opportunity to gauge how well a laboratory is able to sustain, or even extend, quality improvement without the on-site presence of a mentor. These intervals provide an opportunity to evaluate the laboratory’s independent operations and reinforce positive practice and re-address areas of continuing concern. Mentoring programmes should be designed to have a minimum of two engagements per laboratory with a period of four to eight weeks of independent operations between engagements.

### Number of laboratories to be mentored

The number of laboratories to be mentored should be in line with national accreditation priorities. However, this determination should also take into account available human, financial and logistical resources and the guidance above related to sufficient duration and frequency.

### Size of laboratories to be mentored

This can pertain to the number of sections in a laboratory or the size of its staff. Large laboratories with many sections and staff may benefit from a mentoring approach that works on a section-by-section basis, spending several consecutive weeks focusing on each section. In smaller laboratories, a mentor should be able to work across all sections simultaneously, dealing with the laboratory as a whole.

### Number of mentors and their available time

The number of mentors and their availability for deployment is a common limitation confronted when designing mentoring programmes. This can result in tough choices about the mentoring model. When faced with such decisions, it is our view that mentoring programmes should focus on doing more for fewer laboratories.

This may strike some observers as unfair or inadequate to their needs. The argument can be raised that greater reach would enable more laboratories to benefit by receiving at least a little mentoring. While sympathetic to the gap between needs and resources that this view attempts to address, it nonetheless mistakes mentoring to be a commodity that can maintain its value and return on investment while being sub-divided into smaller and smaller units. That is not the case. It has been argued above that the benefits of mentoring accrue through accretion – working side-by-side with the laboratory over an extended time. The benefits realised through mentoring are not accumulated in a linear fashion – spending one week in five labs is not equal to spending five weeks in one lab.

On the surface, the ‘equation’ above may seem like it should be reflexive. But mentoring is about understanding a laboratory’s working culture and helping its primary members re-shape that culture to include the routine implementation of QMS. It relies upon correction, repetition, repeated correction and correct repetition. Because implementation of QMS involves behaviour change, it is often better to provide concentrated mentoring to a few laboratories (along the lines described above) rather than stretch too far, provide diluted mentoring and realise few long-term gains.

Moreover, making the choice to engage a few laboratories more substantively provides the opportunity for greater experiential learning and capacity building of staff working in those laboratories. Laboratory staff who have received in-depth mentoring and subsequently proven their ability to maintain and extend their laboratory’s QMS are prime candidates to implement these same improvements in other laboratories, either on transfer or by themselves serving as mentors. A mentorship design that stretches too wide and has a thin in-lab presence is unlikely to have the same impact in terms of formation of a cadre of valuable managers and/or second-generation mentors.

### Skill level and experience of mentor

Not all mentors will exhibit the same ‘work rate’. This is attributable to differences in skill level, experience, style, acceptability, and the particularities of the individual laboratories. Inexperienced mentors may initially need to spend more time working in laboratories than experienced mentors. National mentors familiar with the intricacies of a country’s laboratory system may find it easier to address certain problems than external mentors who are unsure how or with whom they should work. Mentoring programme design should consider whether inexperienced mentors or mentors unfamiliar with the national laboratory system may need more in-laboratory time.

In addition, mentoring programmes should be attentive to the training needs of the mentors themselves. Regular experience-sharing with other mentors, QMS training, assessing methods of training, ISO 15189 training, QMS implementation study visits – these are some ways in which mentors can continue to build knowledge and skills that can contribute to their in-lab work.

### Funding

Funding considerations contribute significantly to the mentoring model, sometimes necessitating difficult decisions. When faced with limited funds, consider demonstrating success and seeking additional monies to expand, rather than stretching too far and diluting programme impact, thus weakening the case for programme expansion.

### Logistics

Mentors live and move within the areas surrounding the laboratories they work in. Considerations around driving distances and frequencies of flight schedules must also be factored into mentoring design and budgets.

## Mentor support and integration

Technical and logistical support for mentors should also be considered. While the latter can often be addressed through office administrative staff, technical support – as well as integration – may require intentional networking and structure.

Technical support will invariably be needed, as mentors engage the laboratory system’s most deeply rooted problems. Mentors should access technical support from leadership in the MOH and other national experts. For this reason, mentor orientation should include introductions to all relevant resource persons. Organisations that have outside laboratory expertise should link mentors with laboratory specialists and/or experienced mentors working in other settings with whom they can consult as the need arises.

Logistical support ensures that a mentor’s time and energy are primarily directed to laboratories. Because mentors most often work in laboratories rather than an organisation’s office – and may travel extensively – assistance with transportation, accommodation, communication and stationery can be very helpful. The cost of transport, accommodation, meals, phone calls, internet, photocopying, faxing, etc. should be budgeted.

Integration of mentors is important internally, with the MOH, with relevant laboratory working groups or subcommittees and with other laboratory stakeholders. Because mentors often work independently for extended periods, they often operate outside the office or organisational work culture. Specific efforts may be necessary to ensure that mentors are involved and recognised as valuable members of the organisation – for example, scheduling recurrent organisational meetings at times when they can participate. Good mentors can be difficult to replace. Once hired, inclusion and job satisfaction should not be ignored.

If multiple mentors are working in a country, they should meet together routinely to share experiences and synchronise approaches. Mentors should be integrated with the MOH’s national quality department and a representative should participate in national working groups or subcommittees related to QMS implementation or accreditation. This integration will encourage mentor observations from the laboratory level to inform system-level laboratory activities and vice versa.

## Supervision and accountability

Creating an environment that addresses common mentor needs and monitors programme progress enables mentors to focus on in-lab implementation of QMS and accreditation preparedness.

If employing a single mentor, supervision of the mentor may fall to a non-laboratory member of the management team. In-country supervision can assist with the immediate problem-solving needs of both parties. When non-laboratory personnel conduct mentor supervision, it is important to identify outside laboratory technical support to discuss laboratory issues that may arise.

If a programme is designed for a team of mentors, someone should be tasked with coordinating the overall programme and providing supervision. This could be done by a non-lab programme manager or by designating a senior mentor who has reduced in-lab duties and coordinates and supervises the rest of the mentor team. Mentor teams should pursue common objectives in a coordinated manner, within standard structures and with standardised MOH-endorsed tools. While each mentor will have their favoured micro-approaches to working with a laboratory, a structured model, standard tools, and integration will help ensure that laboratories implement QMS in a standard fashion that demonstrates coherence.

Accountability is another crucial element of programme design. Mentors should be clear about to whom and how frequently they report their activities, in what format, what feedback they can expect and when they will receive it. Reporting may include written reports, routine phone calls, regular presentations to the MOH or a relevant working group, etc.

Just as competency assessments are required of all laboratory staff, mentors should also receive field observation as part of their supervision. In addition, mentored laboratories should provide evaluation feedback on mentoring engagements. Follow-up contact with mentored laboratories and/or relevant MOH staff should be regularised to identify any emerging concerns.

Ultimately, laboratory mentoring programmes are accountable to the MOH’s laboratory leadership. As previously stated, the MOH should be included in reporting structures, either directly or through the representation of a mentor coordinator or senior mentor.

## Measuring laboratory progress and mentoring effectiveness

In addition to clearly stated goals, mentoring programmes should also have standard measures of performance in order to gauge laboratory progress and mentoring effectiveness. The regular collection of laboratory performance data using standard tools will indicate how well a laboratory is implementing QMS and where the laboratory stands with regard to their accreditation goals.

The WHO-AFRO SLIPTA program can help in setting clear overall goals and objectives. For example, one mentoring objective may be to realise an improvement of two stars on the five-star SLIPTA scoring scale. Or it may be that all laboratories will operate at the level of at least three stars by the end of the project. Or that a given laboratory will reach five stars and be ready to successfully achieve ISO-15189 accreditation.

The presence of the WHO-AFRO SLIPTA checklist can serve as an important measuring tool since it is standardised and accepted for use across multiple countries. Data collection intervals should be structured to capture information about laboratory performance *during* mentoring engagements as well as *between* mentoring engagements.

It is recommended that assessments be conducted before mentorship begins, at the end of each mentor engagement, when the mentor returns to the laboratory after a period of absence and at the conclusion of mentoring. The importance of the ‘return’ assessment lies in the valuable information it can provide for understanding how well the laboratory is able to sustain QMS implementation under its own strength and initiative.

As with general accreditation preparation, independent assessments conducted by persons familiar with the WHO-AFRO SLIPTA checklist can be an important means of validating progress. Involving independent assessors at baseline and at regular intervals thereafter (e.g., semi-annually or annually) can help identify or confirm general trends and laboratories ready to apply for assessment by WHO-AFRO. Independent assessment at the conclusion of the mentoring programme can aid in measuring overall success.

These on-going assessments serve more than one purpose during the mentorship engagement period. As described above, the assessments at specific time points within the mentorship period provide a means of gauging the laboratory progress, mentoring effectiveness, how well a laboratory is implementing QMS and where the laboratory stands with regard to their accreditation goals. As part of mentorship, these assessments are also a means of building capacity to conduct internal audits within the laboratory as they are jointly done with the quality manager or any of the persons designated to internal audits.

Collection of other performance data is strongly encouraged. Creating a balanced scorecard of key indicators to monitor in mentored laboratories will provide data about the status of service level operations. The granularity and quantitative nature of this monitoring complements the broader evaluation of the WHO-AFRO SLIPTA checklist and ensures that the subjectivity in scoring the SLIPTA assessment is balanced with the presence of performance data.

Mentors arriving at a laboratory for the first time may need to develop a baseline for these indicators by reviewing performance over the last full month or four weeks. This may be difficult in situations where these data may not have been collected or recorded. While collection of these baseline data may be mentor driven, it is important to build this tracking into the monthly operations of the laboratory so that this monitoring becomes a routine laboratory activity. The collection of these data should be standardised across mentored laboratories and could include performance indicators such as turn-around time, service interruptions, stock-outs, equipment downtime, external quality assurance performance, customer complaints and specimen rejection rate.

For ongoing monitoring, laboratory assessments with the WHO-AFRO SLIPTA checklist should continue to be conducted even after the full mentoring engagement has finished. These assessments could be conducted twice a year. If slippage is noted, further mentoring engagement may be prescribed. Incorporating these assessments into the MOH’s evaluation of its laboratories should be strongly considered.

Data summaries should be reported and shared with the MOH and key stakeholders. Efforts should be made to include independent evaluation of mentored laboratories. If a mentoring team is in place, mentors can conduct assessments for laboratories they are *not* mentoring. Another option is to have the senior mentor, a lab specialist or supervisor assess the laboratories, perhaps teamed with an MOH quality officer. The crucial issue is to include assessments of laboratory performance by independent evaluators to promote objectivity and accountability.

## Structured reporting

Reporting mechanisms should allow full engagement of the laboratory staff, laboratory management and upper management, which may include hospital management and the MOH. Any findings and opportunities for improvement must be discussed with the laboratory staff and management and action plans jointly formulated.^4^ Besides guarding against the mentor doing the work for the laboratory, it is one means of building capacity within the laboratory and ensuring sustainability.

Commitment from management is a requirement of ISO 15189,^9^ and constant and persistent engagement of management through proactive reporting is one means of gaining support from them. Management support is critical to the success of implementation of quality management system as significant issues require their direct support, e.g., funds for purchase of reagents and supplies, staffing and physical changes, among many.

## Summary

Laboratory mentorship has been provided over the years in many different formats with varying results.^10,11^ Some achievements have been reported where use was made of short visits, with technical assistance through telephone and SKYPE. The authors observed that there is lack of a documented and standardised or harmonised approach to mentorship. This guide seeks to highlight elements that the authors feel should be considered when setting up a long-term, sustainable mentorship programme. Considerations of these guiding principles may be a step towards harmonising approaches to mentorship. Harmonising the approach may allow scalability and easy comparison across countries. With a standardised approach, budgeting and planning for countries intending to set up mentorship may be easier, as they are able to single out the activities expected.

In conclusion, the success of a mentorship programme depends on several factors: well-defined goals, sufficient length of mentor engagement on site, standardised approach across laboratories, measurement of progress using standardised tools, well-structured reporting mechanisms, alignment of the programme with overall MOH plans, and selection and training of the mentors. These elements will differ in application, depending on countries’ needs and available resources. One to two weeks of mentorship engagements may be sufficient for laboratories that are far advanced in implementation of QMS while six to eight weeks may be required for laboratories that are beginning the process. In some countries where resources to recruit international mentors are not available and local experienced and self-motivated staff may not have the other prerequisites described here, local mentors can be recruited and taken through a grooming phase until they are ready to mentor independently.
